# Biopsy with the New Essen Biopsy Forceps

**DOI:** 10.1155/2013/413259

**Published:** 2013-07-10

**Authors:** Peter G. Traine, Katharina J. E. Schedler, Mariuccia G. S. Brusa, Eduardo B. Rodrigues

**Affiliations:** ^1^Ludwig-Maximilians University, 80331 Munich, Germany; ^2^Macro e Micro Anatomia Patologica, Florianopolis, 88015-120 SC, Brazil; ^3^Department of Ophthalmology, Vision Institute—IPEPO, Federal University of São Paulo, São Paulo, 05599-970 SP, Brazil

## Abstract

*Purpose*. To present initial experience with a novel biopsy method, the Essen biopsy forceps. Therefore, two patients with diagnostic suspicion of uveal melanoma underwent biopsy for histopathological confirmation. *Case Presentation*. Two patients presented with painless unilateral vision reduction. Ultrasound revealed the diagnostic suspicion of uveal melanoma. Therefore, biopsy with the Essen biopsy forceps using a sutureless 23-gauge three-port vitrectomy system was performed. The specimens were then submitted to a pathologist and processed. Histopathology of the obtained specimen confirmed the diagnostic suspicion of choroid melanoma in both patients. *Conclusion*. Essen biopsy forceps is a very practicable alternative method to the FNAB, allowing a combined histopathological and immunohistochemical examination for achieving high diagnostic accuracy at minimal risk.

## 1. Introduction

Intraocular tumors can be diagnosed with clinical exams and additional radiologic tests. In some cases, such as amelanotic melanoma, it is necessary to perform intraocular biopsy. Mostly used method of biopsy is fine-needle aspiration biopsy (FNAB) [1].

The purpose of this paper is to present initial experience with the novel biopsy method, the Essen biopsy forceps (Figure 2(a), Dutch Ophthalmic Research Center, Zuidland, The Netherlands). With this very practicable method, feasible even in smaller medical institutions without direct access to a pathologic department, it is possible to obtain tissue allowing a combined histopathological and immunohistochemical examination.

## 2. Case Reports

### 2.1. Case 1

**Figure 1:**
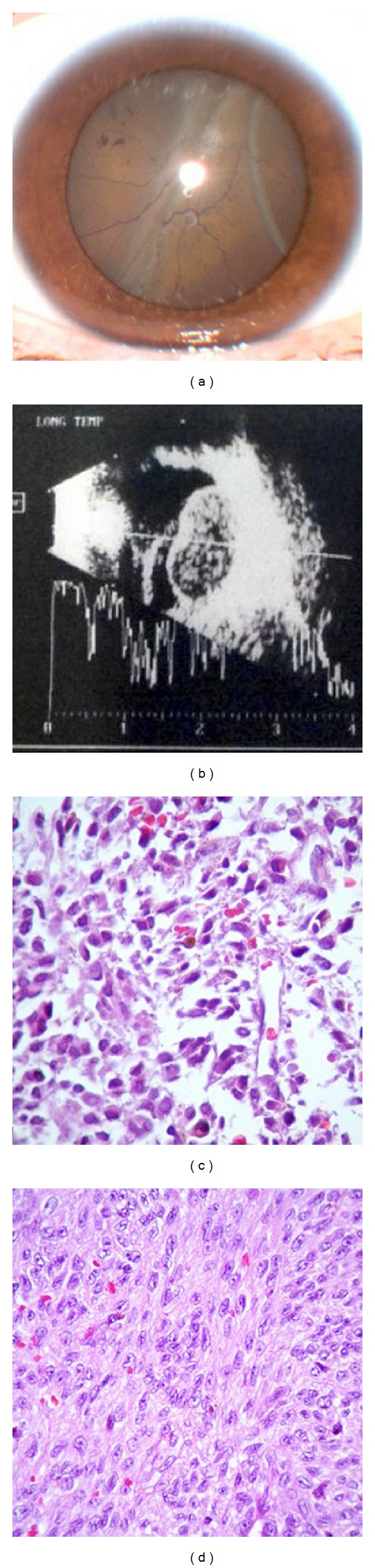
(a) Fundoscopy of the right eye showing a complete retinal detachment and a tumoral mass temporal-inferior-nasal. (b) Ultrasound of the right eye showing a 17 × 15 × 10.5 mm measuring tumoral mass with a positive kappa angle. (c) Biopsy of the right eye, using the Essen biopsy forceps. The specimen obtained measures 2 × 1 mm, and histopathological section shows a fragment of collagenous connective tissue permeated by melanophages and, separate from this, a loose grouping of atypical, polymorphic cells with acidophilic cytoplasm and large, irregular and hyperchromatic nuclei. (d) The histopathology of enucleation showing a 17 × 15 mm spindle cell choroid melanoma (pG1) with areas of ischemic necrosis.

A twenty-three-year old female presented with progressive painless vision reduction on the right eye for 6 months. Her medical, ophthalmological, and family history was unremarkable. She was able to perceive shapes in the right eye and visual acuity was 20/20 in the left eye. Intraocular pressure was 14 and 17 mmHg. Fundoscopy of the right eye showed a complete retinal detachment and a tumoral mass temporal-inferior-nasal (Figure 1(a)). Transpalpebral ultrasonography showed a 17 × 15 × 10.5 mm measuring mass with a positive kappa angle (Figure 1(b)). Suspecting uveal melanoma, the patient requested histopathological confirmation before undergoing enucleation. Biopsy with the Essen biopsy forceps using a sutureless 23-gauge three-port vitrectomy system was performed. The specimen obtained measured 2 × 1 mm, and histopathological section showed a fragment of collagenous connective tissue permeated by melanophages and, separate from this, a loose grouping of atypical, polymorphic cells with acidophilic cytoplasm and large, irregular and hyperchromatic nuclei (Figure 1(c)). These findings confirmed the diagnostic suspicion of uveal melanoma, and two weeks later enucleation was performed. The histopathology of enucleation showed a 17 × 15 mm spindle cell choroid melanoma (pG1) with areas of ischemic necrosis (Figure 1(d)), and pathological staging was pT3a. There were no complications in a ten-month follow-up period.

### 2.2. Case 2

**Figure 2:**
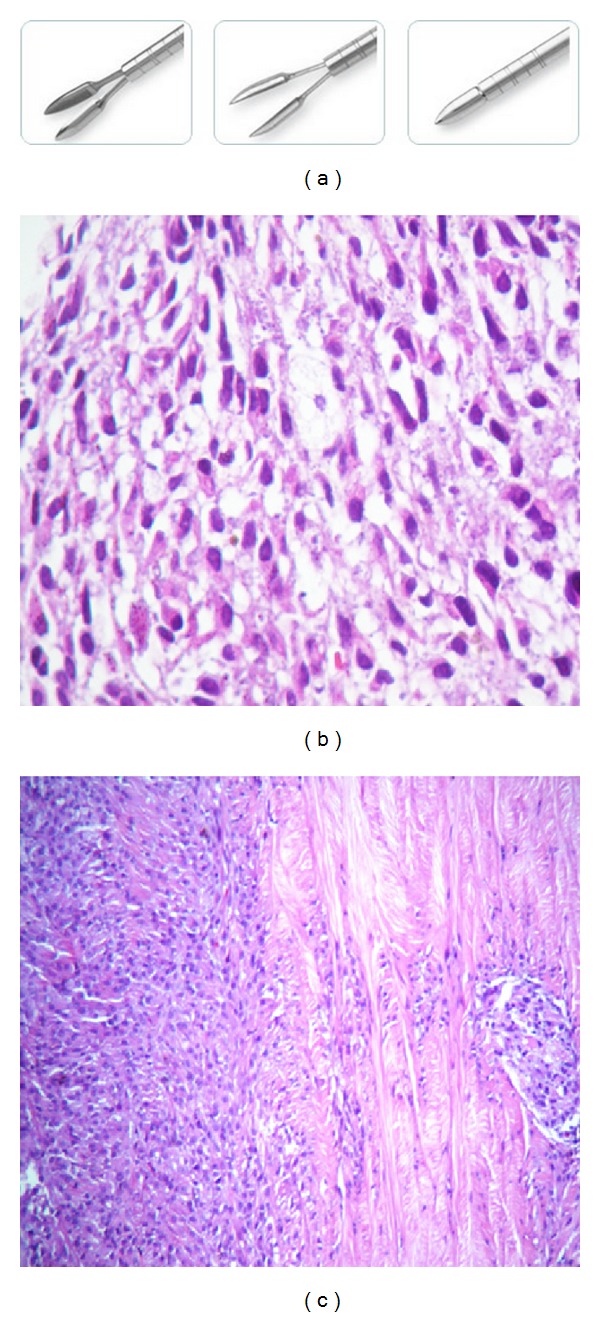
(a) Picture showing the new Essen biopsy forceps. (b) Biopsy of the right eye, using the Essen biopsy forceps. The specimen obtained measures 1 × 1 mm, and histopathological section shows a small fragment of neoplastic tissue formed by conglomeration of cells with acidophilic cytoplasm with poorly defined and atypical nuclei with dense chromatin, sometimes polarized. Some cells containing melanin pigment. (c) Histopathology of enucleation showing a 14 × 13 mm choroid melanoma with areas of recent hemorrhage.

A forty-eight-year old female presented with visual reduction in the right eye for three weeks. Her medical, ophthalmological, and family history was unremarkable. Clinical examination led to the suspicious diagnosis of uveal melanoma. Transpalpebral ultrasonography showed a 17 × 16 × 11 mm measuring tumoral mass with an associated small retinal detachment in the inferior region of the ocular globe. A biopsy using Essen biopsy forceps was offered before performing enucleation. The specimen obtained by the biopsy measured 1 × 1 mm and histopathological section showed a small fragment of neoplastic tissue formed by conglomeration of cells with acidophilic cytoplasm with poorly defined and atypical nuclei with dense chromatin, sometimes polarized. Some cells contained melanin pigment (Figure 2(c)). Histopathology of enucleation showed a 14 × 13 mm choroid melanoma with areas of recent hemorrhage (Figure 2(b)), and pathological staging was pT3a. There were no complications in an eight-month follow-up period.

## 3. Discussion

Biopsy is indicated if diagnostic uncertainty remains, despite previous examinations, which is reported in 2.4% of the cases [2]. A precise diagnosis is important, to distinguish different therapy options such as a specific, invasive, or palliative approach. Some patients demand histopathological confirmation before an enucleation is performed. In uveal melanoma, genetical examinations are getting more important, as they can provide important prognostic information such as metastatic potential of the neoplasia [3]. FNAB, the frequently used method for intraocular biopsy, obtains little specimen and can therefore only be processed for cytological and immunocytological examinations.

In collaboration with the Dutch Ophthalmic Research Center Akgul et al. developed the Essen biopsy forceps as new method for intraocular biopsy [4]. Herein we demonstrate our experience with this method and show its accuracy in providing enough tissue allowing a combined histopathological and immunohistochemical examination. Biopsy, using forceps, is a technically easier procedure than FNAB and in comparison to the latter does not require the presence of a cytopathologist in the operation theater. The specimen is submitted to a pathologist, fixed, and then 6–24 hours later processed. Therefore, biopsy procedure with forceps is a very practicable approach, feasible even in smaller medical institutions without direct access to a pathologic department. 

The risk of tumor cell seeding during this transvitreal approach is expected to be as low as that described for other transvitreal approaches [5, 6]. For evaluating possible side effects and long-term risks like retinal detachment and tumoral seeding an accurate followup and the performance of prospective studies is necessary.

In summary, Essen biopsy forceps is a very practicable alternative method to the FNAB, allowing a combined histopathological and immunohistochemical examination for achieving high diagnostic accuracy at minimal risk.
